# How Increased Dietary Folic Acid Intake Impacts Health Outcomes Through Changes in Inflammation, Angiogenesis, and Neurotoxicity

**DOI:** 10.3390/nu17071286

**Published:** 2025-04-07

**Authors:** Siddarth Gunnala, Lori M. Buhlman, Nafisa M. Jadavji

**Affiliations:** 1 Department of Biomedical Sciences, Midwestern University, Glendale, AZ 85308, USA; 2 College of Osteopathic Medicine, Midwestern University, Glendale, AZ 85308, USA; 3 Department of Biomedical Sciences, School of Medicine, Southern Illinois University, Carbondale, IL 62901, USA; 4 Department of Neuroscience, Carleton University, Ottawa, ON K1S 5B6, Canada; 5 Department of Child Health, College of Medicine—Phoenix, University of Arizona, Phoenix, AZ 85721, USA

**Keywords:** folic acid, over-supplementation, hypoxia, unmetabolized folic acid, ischemic stroke

## Abstract

Dietary folic acid supplementation is well known for playing a crucial role in the closure of the neural tube. Individuals have continued to increase dietary intake of folic acid in counties with mandatory fortication laws in place. Some studies have demonstrated adverse health effects in individuals with high dietary intake of folic acid. Nutrition is a modifiable risk factor for ischemic stroke. Specifically, elevated levels of homocysteine, they can be reduced by increasing intake of vitamins, such as folic acid, a B-vitamin. Hypoxia, when levels of oxygen are reduced, is a major component of cardiovascular diseases. The aim of this review paper was to summarize how increased dietary intake of folic acid interaction with hypoxia to impact health outcomes. Our survey of the literature found that increased dietary intake of folic acid promotes inflammation, angiogenesis, and neurotoxicity. We also report negative actions of increased dietary intake of folic acid with vitamin B12 and genetic deficiencies in one-carbon metabolism. Increased dietary intake of folic acid also results in elevated levels of unmetabolized folic acid in the population, of which the impact on health risks has not yet been determined. Our review of the literature emphasizes that a more comprehensive understanding of the action between increased dietary intake of folic acid on disease outcomes could pave the way for improved public health guidelines. Furthermore, adequate knowledge of an individual’s one-carbon metabolism status can inform proactive management for patients at higher risk of experiencing negative health outcomes.

## 1. Introduction

Nutrition plays a significant role in human health. For example, deficiencies or over-supplementation of vitamins can increase the risk of developing various negative health conditions. There is a strong link between nutrition and increased risk to cardiovascular diseases [1,2,3]. One-carbon (1C) metabolism facilitates nucleotide synthesis and methylation, which are important for cellular function and viability. The functionality of 1C metabolism is dependent on folic acid, as well as cobalamin, riboflavin, and others. Folates are B-vitamins that play a vital role in cellular growth and development [4].

Women of childbearing age that do not have adequate levels of folic acid may have infants with neural tube defects (NTDs) [5,6]. In 1998, both the US and Canadian governments implemented fortification of foods to reduce NTD rates. Other parts of the world have also followed suit like South America. The exact mechanisms through which folic acid reduces NTDs is not well defined [7]. Research done in mice models has shown that increased dietary intake of folic acid per day in adults, can have negative health impacts (1–3). This may result from 1C metabolic pathway dysfunctions, including deficiencies in folic acid receptor functionality, altered enzyme expression, and hidden malnutrition of other essential vitamins associated with 1C metabolism [2,5]. It should be noted that these studies require follow up in humans. Through 1C folic acid plays an important role in nucleotide synthesis, DNA repair, and lipid metabolism. Additionally, folic acid generates methyl groups that aid in the remethylation of homocysteine [8,9,10,11]. Reduced dietary intake of folic acid can increased levels of homocysteine, as there is not enough methyl groups generated to remethylate homocysteine to methionine. It is well established that elevated levels of homocysteine result in increased risk for cardiovascular disease, such as ischemic stroke [12,13,14].

Cardiovascular diseases are a group of disorders that affect the heart and blood vessels. Increased levels of folic acid have been shown to improve endothelial cell function, which is a marker of cardiovascular health [15,16]. The possible mechanisms through which folates improve endothelial cell function have been investigated in experimental models and they include increased antioxidant actions [16], effects on cofactor availability, as well as direct interaction with endothelial nitric oxide synthase (NOS) [17]. Further studies determining the exact mechanisms the impact of increase folic acid levels on endothelial cells are needed in vitro and in vivo, as well as the impact of folic acid on the neurovascular unit.

Levels of methylenetetrahydrofolate reductase (MTHFR) can impact the risk for and outcomes after an ischemic stroke, possibly through levels of homocysteine [1,2,18,19,20]. Reducing levels of homocysteine by increasing dietary levels of folic acid has been demonstrated to be effective [21,22,23]. However, this was not the case initially when clinical trials were conducted to reduce homocysteine levels by supplementation of B-vitamins. The initial evidence showed no benefit of folic acid supplementation to reduce risk or outcomes [24,25,26]. However, further data analysis determined that patients’ renal status was an important factor in consideration of which B-vitamin should be supplemented [3,27,28,29]. A meta-analysis of 82,334 participants showed that the efficacy of folic acid supplementation towards stroke prevention. It was noted that there was a 10% reduction (95% CI 0.84–0.96, *p* = 0.02) in ischemic stroke risk [12]. A study from China, a non-fortified country, demonstrated reduced prevalence of stroke in hypertensive patients treated enlarapil and folic acid (*n* = 10,348 participants, hazard ratio, 0.79, 95% CI 0.68 to 0.93), compared to enlarapil only patients [30].

In preclinical studies a dietary folate deficiency has been reported to result in increased lipids peroixuation and decreased cellular antioxidant defenses [1], furthermore other studies have reported increase neuronal death, DNA damage, and gliosis [2]. Another mechanism in which folates have been reported to have a positive effect on cardiovascular health is ischemic stroke is by lowering levels of homocysteine.

A primary component of cardiovascular diseases, such as ischemic stroke, is the reduction of oxygen levels termed hypoxia. The mechanisms through which increased levels of dietary folic acid impacts health outcomes after hypoxia is poorly understood. The aim of this review is to summarize current proposed mechanisms through which dietary folic acid interacts with hypoxia to impact health outcomes.

## 2. Methods

PubMed, Web of Science, and Google Scholar databases were searched for studies that examined the impact of increased dietary intake of folic acid on outcome after hypoxia. The search terms included a combination of the following words: hypoxia, folic acid, cardiovascular disease, over-supplementation, unmetabolized folic acid, sleep apnea. The year of publication was not limited. Since there are few studies in this area, all studies were included in this review.

## 3. Mechanisms Through Which Folic Acid Acts on Hypoxia to Influence Health Outcomes

### 3.1. Inflammation and Angiogenesis

Hypoxia leads to the initiation of inflammatory responses in common vascular diseases [31]. One of the primary mechanisms by which hypoxia initiates inflammation is through the hypoxia inducible factor-1 (HIF-1) pathway-mediated expression of interleukin-1beta (IL-1β) and tumor necrosis factor-alpha (TNF-α) [31]. TNF-α plays a direct role in the initiation and continuation of inflammation in conditions of hypoxia, while also recruiting other proteins and cells involved in inflammation such as endothelial adhesion molecules and cytokines [31]. IL-1β is a pro-inflammatory cytokine that induces pro-inflammatory gene expression and the synthesis of pro-inflammatory proteins following the initiation of hypoxia associated inflammation via HIF-1α, leading to involvements of leukocytes [31]. Hypoxia reduces IL-10 by three-fold compared to normoxic conditions, which limits and terminates the inflammatory responses via inhibition of activation and effector function of T-cells, macrophages, and monocytes [31].

Optimal folic acid intake for age levels [32] has demonstrated an anti-inflammatory effect through downregulation of IL-1β and TNF-α protein levels [31]. The mechanism is unknown as this is the first evidence of inflammation inhibition by high amounts of folic acid in a THP-1 cell. Interestingly, studies have found that very high levels of folic acid (40 µg/mL) cause inhibition of 1L-10 in cultured human monocytic cells (THP-1) [31]. The underlying mechanism by which this large amount of folic acid causes IL-10 inhibition remains unclear. Vascular endothelial growth factor (VEGF) mRNA levels are decreased in a dose-dependent manner by folic acid treatment [31]. Increased levels of VEGF are typically present during and after hypoxia as part of the HIF-1 pathway which promotes angiogenesis and reperfusion to ischemic tissue. Current studies suggest that folic acid supplementation reduces HIF-1α protein levels in human monocyte cells by attenuating nuclear-cytoplasmic transporter importin α/β in the nucleus [31]. This pioneering study demonstrating a dose-dependent effect of folic acid supplementation on hypoxia induced inflammation will hopefully open the door to additional exploration. Studies exploring the effects of over-supplementation on inflammation and how they interplay with acute/chronic hypoxia in the context of pathologies are warranted. Sequencing and gene expression studies of key genes involved in the hypoxia response cascade and 1C metabolism unique to a variety of in vivo and in vitro models would allow for elucidation of mechanisms by which certain models have robustly adaptive responses to hypoxia, and how some models adapt to aberrant acute and chronic nutritional statuses.

### 3.2. Notch Signaling

Neuronal stem cells (NSCs) are the source for neurogenesis and gliogenesis in the brain [33]. Folic acid regulates neurogenesis and gliogenesis by stimulating the *Notch1* signaling pathway in embryonic NSCs [33]. The *Notch* signaling pathway is vital for NSC proliferation and differentiation; cell surface protein notch 1 is directly involved with the maintenance of NSCs [34]. *Notch* gene expression suppresses apoptosis and promotes proliferation through growth factor-mediated pathways [33]. The *Notch1* signaling pathway includes the hairy and enhancer of split 5 (Hes5) effector, which is integral for differentiation of NSCs [33]. Increasing levels of folic acid mediates proliferation of NSCs via the notch signaling pathway and is directly associated with increasing *Notch1* and *Hes5* mRNA and protein levels [33]. Rats with genetically disrupted *Notch* signaling have increased neuronal differentiation and fewer NSC markers. A study utilizing floating spheroid cell aggregates made from isolated rodent brain NSCs reported a folic acid-mediated increase in *Notch1* and *Hes5* mRNA levels, but intracellular signaling pathways that bridge the influence of folic acid to NSC differentiation mechanisms were not clearly identified [33]. Whether changes in expression of the *Notch* signaling pathway players would be detected in the presence of higher folic acid levels is an open question. Furthermore, 4 mg/L folic acid was delivered to neurospheres in this study, but only 2 mg folic acid/kg diet was delivered to maternal rats in a referenced study that was observing cell proliferation and apoptosis in fetal mice brains associated with folic acid deficiencies [34]. Through 1C folic acid promotes DNA methylation. This may be the mechanism by which folic acid acts on NSCs [34].

### 3.3. Neurotoxicity of Folic Acid

Aberrant folic acid supplementation has been primarily shown to involve folic acid receptor and enzyme dysfunction. Folic acid is transported around the body via the use of a variety of folate receptors; an abnormality caused by pathology or genetic mutation in these can lead to a bottleneck in 1C metabolism and exacerbate detriments of increased levels. Deficits of enzymes involved in folic acid metabolism can also cause pathologies from increased levels of folic acid because of changes in levels of 1C metabolism intermediates and their receptors. Effects of excess folic acid in 1C metabolism can elicit neurotoxicity. It is like what is caused by excess kainic acid (KA), a powerful analogue of the neurotransmitter glutamate that is involved in neurotoxic glutamatergic postsynaptic receptor excitation [35]. Methyl-tetrahydrofolate (MTHF), a derivative of folic acid, competes with KA for binding sites on rat cerebellar membranes [35]. N-5-formyltetrahydrofolate (FTHF) is another folic acid derivative that is more potent than MTHF regarding limbic seizure production and subsequent brain damage [35]. Seizure-like behavioral symptoms and cytopathological changes like those produced by KA dosing can be elicited by FTHF and folic acid application at half the dose of MTHF [35]. Polyglutamic acid (PGA) and FTHF produce distinct patterns of KA-like neuronal damage and seizure-like symptoms, but lack the novel damage caused at the site of injection the way that KA does [35]. Preapplication of DHFR blocker amethopterin to the amygdala does not reduce KA-like neurotoxicity or seizure-like behavior [35]. Thus, neurotoxic levels of PGA appear to be independent of FTHF. Taken together, exposure to high levels of folic acid may induce convulsion, and folic acid metabolites may be directly implicated in epilepsy and related brain damage.

### 3.4. Unmetabolized Folic Acid

Folic acid metabolites have several destinations, such as storage in tissue, secretion into bile, reabsorption and metabolization by the liver, or excretion by the kidneys [36]. Initial metabolism of folic acid in the liver from the intestines is facilitated by high liver expression of DHFR compared to the rest of the body [36]. Because of the increased capacity of folic acid reduction in the liver, unmetabolized folic acid (UMFA) does not accumulate during folate digestion but is directly related to fasting state. The efficacy of folic acid absorption is dependent on multiple additional factors, such as changes to the intestinal pH, and the consumption of drugs or alcohol [36].

Very high doses of folic acid, those around 10 to 20-fold higher than what is required for rodents, cause growth retardation in newborn rodents [36]. Increased dietary intake of folic acid leading to similar relative levels of UMFA is becoming more common in human populations. Some countries such as the US have been participating in folic acid supplementation programs, and the list of participating countries has increased to approximately 70 which recommend a minimum additional intake of 150–200 µm of folic acid per day [36]. Higher UMFA serum concentrations have been detected in older individuals who have shorter fasting times. The National Health and Nutrition Examination Survey found serum concentrations of more than 1 nmol/L UMFA in 33% of samples, and in 36% when observing those over the age of 60 years [3]. Some studies have identified an improvement of cognition with higher levels of 5-MTHF but also state that higher supplementations of folic acid causing elevated unmetabolized serum folic acid levels lead to poorer cognitive function [37]. Other evidence shows that UMFA may reduce immune function [4]. One of the primary drivers of this phenomenon is believed to be due to shared biochemical pathways between folic acid and vitamin B12, causing homocysteine accumulation and diminished methylation potential [37]. Deficiencies in micronutrients like those of the folate cycle (e.g., methylcolbamin and pyridoxine 5-phosphate) are common in older populations and could influence UMFA plasma levels [36]. The recycling of folic acid within the body depends on vitamins B12 and B6 [36]. Thus, use of a B-complex supplement versus independent B12 reduces the prevalence of UMFA [36]. Non-supplement users may have higher serum increases in UMFA after fortification compared to those that use multivitamins [36], further demonstrating the importance of B-complex multivitamin supplementation rather than use of independent B vitamin supplementation. More investigation of high UMFA levels on health outcomes and mechanisms is needed so that better informed decisions can be made about dietary folic acid supplementation.

### 3.5. Vitamin B12 and Folic Acid Actions

Vitamin B12 plays a critical role in the recycling and regulation of 1C metabolism [36]. The involvement of vitamin B12 as a coenzyme promotes lipid metabolism, methylation, and DNA formation during cell division [37]. Vitamin B12 is necessary for vascular health, cognitive function, red blood cell synthesis, and lowering the risk of neural tube defects [38]. Macrocytic anemia associated with B12 deficiency is masked by folic acid over-supplementation [38]. In fact, populations that are deficient in B12 and over-supplemented in folic acid are about twice as likely to become anemic compared to populations that are deficient in B12 with therapeutic folic acid levels [38]. Deficiencies in B12 cause depletion of intracellular folates concentrations in rat liver and human erythrocytes [39]. As a coenzyme involved in L-methylmalonyl-CoA isomerization, B12 deficiency leads to elevated methylmalonic acid (MMA) serum and urine levels, making MMA an ideal biomarker for B12 deficiency [39]. MMA has also been identified as a neurotoxic organic acid that can cause dysfunctional myelination in neurons [37].

Vitamin B12 deficiencies have been linked to brain-related disorders, such as neuropathy, neuropsychiatric disorders, cognitive impairment, and reversible dementia [39]. Vitamin B12 deficiency can trap folic acid metabolites, such as 5-MTHF, and therefore inhibit the re-methylation of homocysteine and cause pathologies associated with decreased synthesis of methylation cycle metabolites [39]. Decreased homocysteine remethylation and potential homocysteinemia caused by B12 deficiencies may lead to megaloblastic anemia, neuropathy, and neuropsychiatric disorders [39]. While folic acid has a relatively low potential for supplemental toxicity, large amounts of folic acid may mask vitamin B12 deficiencies in cases of pernicious anemia, presumably since folic acid improves hematological but not neurological indices [39]. Individuals with normal vitamin B12 levels and elevated folic acid levels may be at an increased risk of cognitive impairment due to the accumulation of homocysteine or lowered methylation potential [37].

Vitamin B12 deficiency decreased hepatic folates, but serum folates were relatively unaffected in animals administered normal folic acid supplementation [39]. This phenomenon is attributed to ‘methylfolate trapping’ caused by a B12 coenzyme deficiency leading to impaired intracellular storage of folates [39]. Thus, vitamin B12 deficiencies can lower hepatic folates levels and exacerbate metabolic dysfunction associated with folic acid and 1C metabolism. Under conditions of normal vitamin B12 levels, rat plasma folic acid levels are more affected than hepatic folic acid storage levels by folic acid over-supplementation [39]. In Table 1, we have summarized the impact of increased dietary folic acid levels on deficient and adequate levels of vitamin B12.

### 3.6. Genetic Deficiencies in 1C Metabolism

The propensity for stroke increases with factor of age, and stroke risk increases with dysregulated expression of genes involved in maintenance of metabolic processes [1]. The expression of genes over the course of several cycles of mitotic replication is an intricate process involving DNA methylation [40]. Metabolites from folic acid 1C metabolism, especially tetrahydrofolate (THF), are involved in DNA synthesis, methionine synthesis, transulfuration, and polyglutamate deposition. DNA methylation is necessary for epigenetic regulation of tissue specific gene expression [40].

Genetic deficiencies that affect folic acid metabolism contribute to an increased risk and worse outcome after ischemic stroke [1]. MTHFR typically catalyzes the irreversible conversion of 5,10 methylenetetrahydrofolate (5,10-MTHF) to 5-methyltetrahydrofolate (5-MTHF) [1]. Homocysteine is typically methylated from 5-MTHF with the assistance of vitamin B12 and methionine synthase in the methionine cycle. Thus, populations with the MTHFR 677TT genetic polymorphism have increased plasma levels of homocysteine [1].

Decreased liver MTHFR protein levels and short-term memory impairment can be observed in pups of mice placed on high folate diets. While decreased short-term memory was not observed in pregnant mice, lactating mice also had lower MTHFR levels in the liver [41]. Reduction of MTHFR levels leads to brain homocysteine accumulation, which precedes an increase in apoptotic events and can manifest as impaired motor function and short-term memory deficits [42]. Reduced MTHFR protein levels are common in aged populations and may lead to cognitive deficits [42].

Lower folic acid enzyme levels are implicated in the pathologies of cancer. Folic acid metabolism enzyme methylenetetrahydrofolate dehydrogenase 2 (MTHFD2) activity is correlated with cell proliferation rates in several cancers [43]. The role of MTHFD2 in glioma is controversial, as studies claim its presence or absence [28]. Some studies propose that the development of cancer can reactivate embryogenesis-specific enzymes, such as MTHFD2, for which levels have been shown to drop significantly after birth [43]. Considering the activity of folic acid in the brain, studies exploring the effect of folic acid over-supplementation on MTHFD2 and other 1C metabolism players in the context of brain cancers like glioblastoma are warranted.

### 3.7. Sleep Apnea and Folic Acid Metabolism

Obstructive sleep apnea (OSA) is a breathing disorder characterized by cyclic upper airway closure leading to limitations of airflow while sleeping [44]. OSA frequently leads to the development of cognitive deficits and CVD, for which hypoxia is one of the primary underlying mechanisms [45]. Folic acid is involved in antioxidant pathways that play an integral role in the promotion and prevention of cognitive decline and CVD leading to OSA [45]. As folic acid supplementation is vital for maintaining cognitive function and vascular organ health, detriments of 1C metabolism caused by increased dietary intake of folic acid can hinder control of the inflammatory response leading to higher prevalence and severity in pathologies associated with hypoxia induced inflammation [31]. In studies utilizing THP-1 cells, treatment with intermittent hypoxia with re-oxygenation resulted in hypomethylation of formyl peptide receptor 1 (FPR-1) and hypermethylation of FPR-2 and FPR-3 [44]. FPRs 1/2/3 are G protein-coupled pattern recognition receptors expressed by mammalian leukocytes [44]. FPR-3 is involved in pathogen recognition, FPR-1 is involved in chemotaxis and reactive oxygen species production, and FPR-2 accelerates the resolution of inflammation [44]. Dysregulation of methylation of these genes may lead to the development of OSA, especially in populations at higher risk with CVD. Current studies surrounding the impact of nutrition on antioxidant pathways are sparse and only discuss the detriments of under supplementation. A better understand of the mechanisms by which folic acid over-supplementation potentially leads to decreased 1C metabolism enzyme activity may implicate over supplementation in the development of OSA, resulting from DNA methylation discrepancies associated with improper responses to hypoxia. This aberrancy in DNA methylation has an indirect but significant potential impact on cardiovascular disease and its increasing occurrence.

## 4. Discussion

Studies have demonstrated adverse health effects in individuals with high dietary intake of folic acid [46,47]. The aim of this review paper was to summarize how increased dietary intake of folic acid interaction with hypoxia to impact health outcomes. Increased dietary intake of folic acid over promotes inflammation, angiogenesis, and neurotoxicity, also results in elevated levels of unmetabolized folic acid in the population. We also report negative actions of increased dietary intake of folic acid with vitamin B12 and genetic deficiencies in 1C metabolism.

The present review emphasizes that a more comprehensive understanding of the action between increased dietary increased levels of folic acid on disease outcomes could pave the way for improved public health guidelines. Furthermore, adequate knowledge of an individual’s 1C metabolism status can inform proactive management for patients at higher risk of experiencing negative health outcomes. Studies regarding folic acid metabolism lack adequate focus on the intermediate metabolites of the 1C pathway [48]. Future research should compare results from folic acid studies performed in countries that partake in or abstain from mandatory folic acid fortification programs [49]. Current folic acid studies involving populations lack comprehensive data on dietary habits outside of certain food groups associated with folic acid. Hydration status, family history, medication history, and stress questionnaires are all potential ways to develop a better understanding of changes in the intake and metabolism of folic acid [50]. Genetic testing for predispositions to nutritional sensitivities could also make questionnaire data more robust and even highlight certain demographics of people that are at a higher risk for exacerbation of folic acid over-supplementation pathogenesis. The preservation of homeostasis through efficient cell growth and turnover is a vital function of 1C metabolism [51]. Thus, the need to address the overall health of the individual when studying the metabolism of folic acid in a clinical setting is important, especially with considering variance of activity for folates receptors and enzymes, nutritional conditions, and the presence of other pathologies.

There is still a lot of research required in this area. Mechanistic understanding of how increased levels of UFMA impact health outcomes is needed. A study examining cardiovascular disease outcomes in fortified versus non-fortified countries is needed. There are predictions that the benefit of increasing dietary folic acid intake in non-fortified countries would have more beneficial effects [5].

## 5. Conclusions

As precision medicine continues to develop momentum, it allows for a thorough understanding of the genetic rationale behind pathological phenomena. Understanding what happens in conditions of overnutrition has not been a priority outside of the context of obesity-related disorders.

Adequate understandings of 1C metabolism condition in an individual can inform prophylactic management of stroke for demographics that are at higher risk. Individualized measurements could include genetic testing for common polymorphisms in enzymes involved in 1C (e.g., MTHFR), plasma homocysteine levels, methylmalonic acid in blood or urine. This can be combined with other therapies to further improve the quality of life in at-risk individuals and improve overall health. A baseline understanding of how stroke outcome and recovery are directly affected by folic acid levels can lead to studies of how the prototypical response to hypoxia by folic acid levels is additionally affected by modulations of frequency and intensity in stroke.

## Figures and Tables

**Table 1:** Health outcomes summarized when dietary intake of folic acid is high and vitamin B12 levels are either deficient or adequate.

Vitamin B12 Intake	Health Outcomes
Deficient	Results in methyl trap. Negative health otucomes, including megaloblastic anemia, neuropathy, reversible dementia, neuropsychiatric diseases, and cognitive impairment.
Adequate	Changes in hepatic storage of folic acid and risk of cognitive impairment.

## Abbreviations

The following abbreviations are used in this manuscript:

1C	One-Carbon Metabolism
HIF-1	Hypoxia inducible factor-1
IL-1β	interleukin-1beta
MTHFR	methylenetetrahydrofolate reductase
TNF-α	tumor necrosis factor-alpha
